# Orphan Nuclear Receptor ERRγ Is a Transcriptional Regulator of CB1 Receptor-Mediated TFR2 Gene Expression in Hepatocytes

**DOI:** 10.3390/ijms22116021

**Published:** 2021-06-02

**Authors:** Bo-Eun Kim, Byungyoon Choi, Woo-Ram Park, Yu-Ji Kim, In-Young Kim, Yoon Seok Jung, Yong-Hoon Kim, Chul-Ho Lee, Hueng-Sik Choi, Don-Kyu Kim

**Affiliations:** 1 Department of Integrative Food, Bioscience and Biotechnology, Chonnam National University, Gwangju 61186, Korea; pselh5803@naver.com (B.-E.K.); wolfchoi1035@naver.com (B.C.); dnfka9210@naver.com (W.-R.P.); call7502@naver.com (Y.-J.K.); dlsdud2044@naver.com (I.-Y.K.); 2 School of Biological Sciences and Technology, Chonnam National University, Gwangju 61186, Korea; yhemm@naver.com (Y.S.J.); hsc@chonnam.ac.kr (H.-S.C.); 3 Laboratory Animal Resource Center, Korea Research Institute of Bioscience and Biotechnology, Daejeon 34141, Korea; yhoonkim@kribb.re.kr (Y.-H.K.); chullee@kribb.re.kr (C.-H.L.); 4 Department of Functional Genomics, KRIBB School of Bioscience, Korea University of Science and Technology (UST), Daejeon 34141, Korea

**Keywords:** estrogen-related receptor γ (ERRγ), transferrin receptor 2 (TFR2), cannabinoid receptor type 1 (CB1), orphan nuclear receptor, gene regulation, 2-AG, hepatic iron overload, alcoholic liver disease

## Abstract

Orphan nuclear receptor estrogen-related receptor γ (ERRγ) is an important transcription factor modulating gene transcription involved in endocrine control of liver metabolism. Transferrin receptor 2 (TFR2), a carrier protein for transferrin, is involved in hepatic iron overload in alcoholic liver disease (ALD). However, TFR2 gene transcriptional regulation in hepatocytes remains largely unknown. In this study, we described a detailed molecular mechanism of hepatic TFR2 gene expression involving ERRγ in response to an endocannabinoid 2-arachidonoylglycerol (2-AG). Treatment with 2-AG and arachidonyl-2′-chloroethylamide, a selective cannabinoid receptor type 1 (CB1) receptor agonist, increased ERRγ and TFR2 expression in hepatocytes. Overexpression of ERRγ was sufficient to induce TFR2 expression in both human and mouse hepatocytes. In addition, ERRγ knockdown significantly decreased 2-AG or alcohol-mediated TFR2 gene expression in cultured hepatocytes and mouse livers. Finally, deletion and mutation analysis of the TFR2 gene promoter demonstrated that ERRγ directly modulated TFR2 gene transcription via binding to an ERR-response element. This was further confirmed by chromatin immunoprecipitation assay. Taken together, these results reveal a previously unrecognized role of ERRγ in the transcriptional regulation of TFR2 gene expression in response to alcohol.

## 1. Introduction

Endocannabinoid signaling is an important physiological system that can affect obesity and metabolic syndrome, including chronic pain, liver fibrosis, and a number of inflammatory vaginal and allergic conditions [1]. The endocannabinoid system includes two G protein-coupled receptors: cannabinoid receptor type 1 (CB1) and type 2 (CB2) [1]. The CB1 receptor is expressed in the heart, brain, vascular tissue, and liver, while the CB2 receptor is expressed primarily in cells of immune and hematopoietic systems [2]. It was reported that endocannabinoids, anandamide, and 2-arachidonoylglycerol (2-AG) are associated with neuronal, immune, metabolic, vascular, and reproductive functions. Synthesis of 2-AG is achieved through hydrolysis of diacylglycerol (DAG) by alcohol-induced DAG lipases. It activates hepatic CB1 receptor signaling [3,4,5]. Cytochrome P450 2E1 (CYP2E1) is known to be a major enzyme involved in alcohol-induced generation of reactive oxygen species (ROS) that can lead to oxidative stress and liver injury [6,7]. The pathway involving alcohol-induced hepatic CYP2E1 expression contains the CB1 receptor and estrogen-related receptor γ (ERRγ). Alcohol-mediated activation of the CB1 receptor can increase hepatic ERRγ expression, which in turn triggers CYP2E1-mediated ROS generation and liver damage [8]. In addition, alcohol-mediated activation of CB1 receptor signaling can induce ERRγ-mediated alcoholic fatty liver [9]. These findings indicate that the CB1 receptor plays a critical role in the pathogenesis of alcoholic liver disease (ALD) including alcoholic fatty liver, liver injury, and liver fibrosis [2].

Transferrin receptors (TFR1 and TFR2) are carrier proteins for transferrin. They play an important role in the import of iron into cells by internalizing iron-load transferrin [10,11]. TFR1 is expressed evenly on the surface of normal cells, especially on the surface of maturing erythroblasts, while TFR2 is expressed mainly in the liver [12]. TFR2 expression is positively regulated by GATA binding protein 1, Kruppel-like factor, and CCAAT enhancer binding protein α in mouse embryonic fibroblast cells [13]. Recently, it was reported that orphan nuclear receptor hepatocyte nuclear factor 4α (HNF-4α) is a transcriptional regulator of hepatic TFR2 expression, as demonstrated in liver-specific HNF-4α-null mice [14]. In addition, the TFR2 is involved in hepatic iron overload and hereditary hemochromatosis, as shown in different TFR2-deficient mouse models including whole-body and liver-specific TFR2 knockout (KO) mouse and TFR2/HFE double KO mouse [15,16,17]. Given that TFR2 has less affinity to holo-transferrin than TFR1 [18], the TFR2 is considered as an iron senser. This notion is further supported by a previous report showing that the TFR2 can increases gene transcription of hepcidin, a key iron-regulatory hormone secreted from the liver [19]. Hepcidin can bind to ferroportin (an iron exporter) and induce its internalization and lysosomal degradation, leading to a decreased efflux of iron from duodenal enterocytes [20]. Interestingly, it was reported that the hepatic iron overload has been observed in human alcoholics [21]. More recently, it was demonstrated that hepatic TFR expression is upregulated in human patients with ALD and that such upregulation is associated with hepatic iron overload [22]. However, the endocrine signaling and transcription factor that cause hepatic iron overload in ALD remain largely unknown.

ERRs (ERRα, β and γ) belong to the NR3B subfamily of the nuclear receptor superfamily. They are classified as orphan nuclear receptors because their endogenous ligands have not been clearly identified yet [23]. Interestingly, ERRγ is considered to be constitutively active because its ligand-binding domain (LBD) can adopt a transcriptionally active conformation in the absence of a ligand [24]. ERRγ transcriptional activity is mainly modulated by interaction with co-regulators, such as peroxisome proliferator-activated receptor γ coactivator-1α (PGC-1α) and small heterodimer partner, and post-translational modifications, such as phosphorylation and glycosylation [25,26,27,28]. ERRγ is known to be expressed mainly in key metabolic tissues such as adipose tissue, muscle, and liver [23]. Interestingly, ERRγ expression is inducible in the liver and tightly controlled by multiple endocrine and metabolic signals such as hypoxia, endocannabinoid, insulin, and glucagon [23]. Previously, we revealed that ERRγ is a critical transcriptional regulator of hepatic lipid and alcohol metabolism as well as iron metabolism based on in vitro and in vivo studies [23]. For example, ERRγ can increase the gene expression of CYP2E1 and sterol regulatory element-binding protein-1c, resulting in ALD such as hepatic steatosis and liver injury [8,9]. Furthermore, ERRγ controls systemic iron homeostasis by inducing hepcidin expression [29]. Considering hepatic iron overload in human patients with ALD [22], ERRγ might function as a molecular link between endocannabinoid signaling and hepatic iron overload in ALD. Therefore, the objective of this study was to investigate the role of ERRγ as a key transcriptional mediator in CB1 receptor-induced TFR2 expression in hepatocytes.

## 2. Results

### 2.1. CB1 Receptor Signaling Increases ERRγ and TFR2 Gene Expression in Hepatocytes

**Figure 1:**
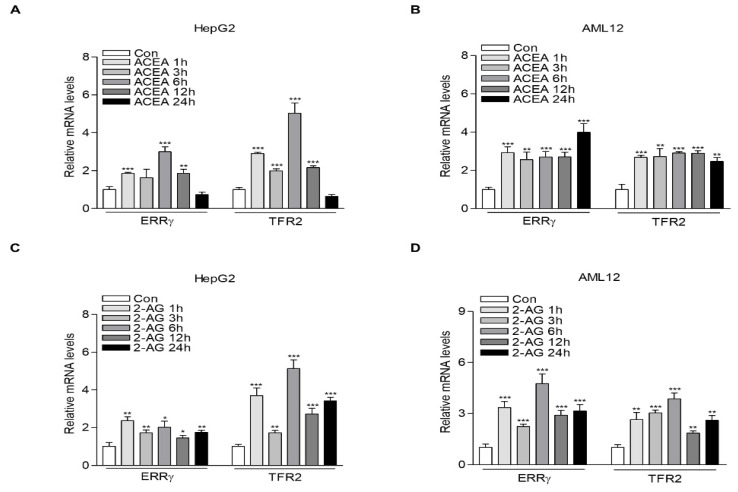
CB1 receptor signaling induces ERRγ and TFR2 mRNA expression. (**A**,**B**) Q-PCR analysis showing ERRγ and TFR2 mRNA levels. HepG2 (**A**) and AML12 (**B**) cells were treated with ACEA (10 μM) for the indicated time. (**C**,**D**) Q-PCR analysis showing ERRγ and TFR2 mRNA levels. HepG2 (**C**) and AML12 (**D**) cells were treated with 2-AG (10 μM) for the indicated time. All experiments were performed in triplicate and repeated at least three times. Error bars show ± SD. * *p* < 0.05, ** *p* < 0.01, *** *p* < 0.001 by two-tailed Student’s *t*-test.

In an attempt to examine whether CB1 receptor signal might be involved in the upregulation of TFR2 implicated in hepatic iron overload [22], we analyzed the mRNA levels of TFR2 in HepG2, a human hepatoblastoma-derived cell line, and AML12, a mouse hepatocyte cell line after treatment with ACEA, a selective CB1 receptor agonist, in a time-dependent manner. Interestingly, TFR2 mRNA levels were markedly enhanced only at 1 h after ACEA treatment, reaching the maximum level at 6 h in HepG2 cells (Figure 1A). However, in AML12 cells, TFR2 mRNA levels were increased within 1 h after treatment with ACEA. Their levels remained elevated until 24 h (Figure 1B). Furthermore, we found that the expression patterns of TFR2 by CB1 receptor signal were similar to those of ERRγ, a critical transcriptional mediator of multiple endocrine and metabolic signals in the liver [23]. Therefore, we examined ERRγ and TFR2 mRNA levels in HepG2 and AML12 cells treated with 2-AG, an endocannabinoid, in a time-dependent manner. As a result, both ERRγ and TFR2 mRNA levels were significantly increased at 1 h after 2-AG treatment. Their levels remained elevated until 24 h in both the HepG2 and AML12 cells (Figure 1C,D).

**Figure 2:**
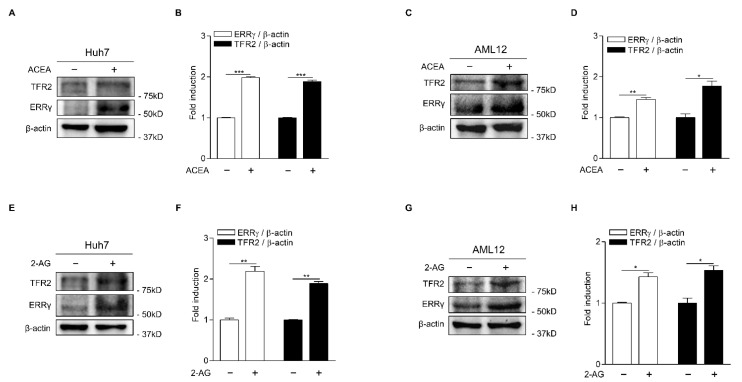
CB1 receptor signaling induces ERRγ and TFR2 protein expression. (**A**–**D**) Western blot analysis (**A**,**C**) and graphical representation (**B**,**D**) showing ERRγ and TFR2 protein levels. Huh7 cells (**A**) and AML12 cells (**C**) were treated with ACEA (10 μM) for 12 h. (**E**–**H**) Western blot analysis (**E**,**G**) and graphical representation (**F**,**H**) showing ERRγ and TFR2 protein levels. Huh7 cells (**E**) and AML12 cells (**G**) were treated with 2-AG (10 μM) for 12 h. The independent experiments were repeated at least twice. Error bars show ± SD. * *p* < 0.05, ** *p* < 0.01, *** *p* < 0.001 by two-tailed Student’s *t*-test.

To test if CB1 receptor signal could lead to the induction of ERRγ and TFR2 protein, we performed Western blot analysis in Huh7, another human hepatoma cell line, and AML12 cells treated with ACEA. As expected, ACEA treatment significantly increased ERRγ and TFR2 protein levels in both Huh7 and AML12 cells (Figure 2A–D). Treatment with 2-AG also significantly promoted the induction of ERRγ and TFR2 proteins in Huh7 and AML12 cells (Figure 2E–H). These findings suggest that CB1 receptor signal is implicated in the transcriptional regulation of TFR2 in hepatocytes.

### 2.2. ERRγ Increases TFR2 Gene Expression

**Figure 3:**
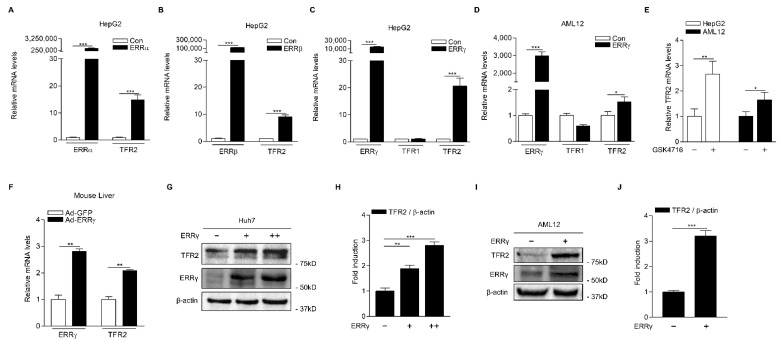
ERRγ increases TFR2 mRNA and protein levels. (**A**–**C**) Q-PCR analysis showing ERRγ, TFR1, and TFR2 mRNA levels. HepG2 cells were transfected with vectors expressing ERRα (**A**), ERRβ (**B**), and ERRγ (**C**) for 48 h. (**D**) Q-PCR analysis showing ERRγ, TFR1, and TFR2 mRNA levels. AML12 cells were transfected with vector expressing ERRγ for 48 h. (**E**) Q-PCR analysis showing TFR2 mRNA levels. HepG2 cells and AML12 cells were treated with GSK4716 (10 μM) for 12 h. (**F**) Q-PCR analysis showing ERRγ and TFR2 mRNA levels in the liver. Ad-GFP and Ad-ERRγ were injected via tail veins into male C57BL/6J mice (*n* = 3 per group). Mice were then sacrificed at day 6. (**G**,**H**) Western blot analysis (**G**) and graphical representation (**H**) showing TFR2 protein levels. Huh7 cells were transfected with vector expressing ERRγ (+: 1 μg, ++: 3 μg) for 48 h. (**I**,**J**) Western blot analysis (**I**) and graphical representation (**J**) showing TFR2 protein levels. AML12 cells were transfected with a vector expressing ERRγ (+: 3 μg) for 48 h. Experiments were performed in duplicate (**G**–**J**) or triplicate (**A**–**F**) and repeated at least twice. Error bars show ± SD. * *p* < 0.05, ** *p* < 0.01, *** *p* < 0.001 by two-tailed Student’s *t*-test.

Previously, we reported that ERRγ is a transcriptional regulator of hepatic CB1 receptor involved in alcoholic fatty liver and oxidative liver injury [8,9]. Based on results showing CB1 receptor-mediated induction of ERRγ and TFR2 genes, we examined whether TFR2 gene transcription might be regulated by ERR isoforms (ERRα, ERRβ and ERRγ) in hepatocytes. Interestingly, overexpression of ERR isoform significantly increased TFR2 mRNA levels in HepG2 cells (Figure 3A–C). Since ERRγ resulted in the strongest induction of TFR2 mRNA levels (Figure 3C), we further examined the effect of ERRγ on TFR2 gene expression in AML12 cells. As expected, overexpression of ERRγ significantly increased TFR2 mRNA levels in this mouse cell line (Figure 3D). However, we did not observe any significant changes in TFR1 mRNA levels in HepG2 cells and AML12 cells transfected with ERRγ (Figure 3C,D). Moreover, GSK4716, a selective ERRβ/γ agonist, significantly increased TFR2 expression in HepG2 and AML12 cells (Figure 3E) [30]. We also confirmed that TFR2 mRNA expression was significantly induced in livers of mice injected with ad-ERRγ (Figure 3F). TFR2 protein levels were also highly induced in both Huh7 and AML12 cells transfected with a vector expressing ERRγ (Figure 3G–J). These results suggest that ERRγ is an upstream transcriptional regulator of TFR2 gene expression in hepatocytes.

### 2.3. Knockdown of ERRγ Decreases CB1 Receptor-Mediated TFR2 Expression

**Figure 4:**
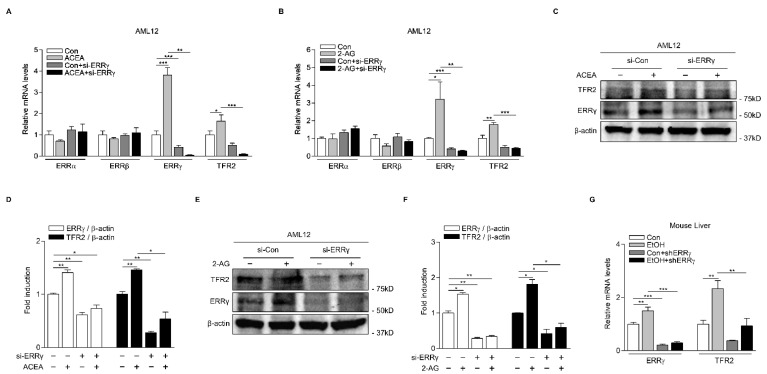
Knockdown of ERRγ decreases CB1 receptor-mediated TFR2 gene expression. (**A**,**B**) Q-PCR analysis showing ERRγ knockdown effect on CB1 receptor-mediated TFR2 mRNA levels. AML12 cells were transfected with si-Con or si-ERRγ for 48 h and then treated with either ACEA (10 μM) (**A**) or 2-AG (10 μM) (**B**) for 12 h. (**C**–**F**) Western blot analysis and graphical representation showing ERRγ and TFR2 protein levels. AML12 cells were transfected with si-Con or si-ERRγ for 48 h and then treated with ACEA (10 μM) (**C**,**D**) and 2-AG (10 μM) (**E**,**F**) for 12 h. (**G**) Ad-US or Ad-shERRγ were injected via tail veins of male C57BL/6J mice (*n* = 5 per group). At day 6 after the injection, mice were treated with vehicle or alcohol (6 g/kg) for 24 h. Q-PCR analysis showing mRNA levels of ERRγ and TFR2 in livers of mice. All experiments were performed in duplicate (**C**–**F**) or triplicate (**A**,**B**,**G**) and repeated at least twice. Error bars show ± SD. * *p* < 0.05, ** *p* < 0.01, *** *p* < 0.001 by two-tailed Student’s *t*-test.

To further demonstrate the role of ERRγ in CB1 receptor-mediated TFR2, we performed loss-of-function studies both in vitro and in vivo. As expected, ACEA treatment significantly increased ERRγ and TFR2 mRNA levels, while such increases were almost entirely blocked by ERRγ knockdown using small interfering RNAs for ERRγ (si-ERRγ) in AML12 cells (Figure 4A). Consistent with these findings, ERRγ knockdown significantly attenuated basal and 2-AG-induced TFR2 gene expression levels (Figure 4B). However, we did not observe any significant change in the ERRα and ERRβ mRNA levels in AML12 cells treated with ACEA or 2-AG (Figure 4A,B). In addition to gene transcription analysis of ERRγ and TFR2, we also carried out Western blot analysis to examine their protein expression levels in AML12 cells treated with ACEA and 2-AG. As a result, ERRγ and TFR2 protein levels induced by ACEA were inhibited by ERRγ knockdown (Figure 4C,D), consistent with results of their mRNA levels. Moreover, 2-AG treatment significantly induced ERRγ and TFR2 protein levels. Such increases were almost completely blocked by ERRγ knockdown (Figure 4E,F). It has been reported that 2-AG synthesis is through alcohol-mediated induction of hepatic DAG lipase, a 2-AG biosynthetic enzyme [3,4,5]. Therefore, we examined the role of ERRγ in alcohol-induced hepatic TFR2 mRNA expression. Modeling hepatic ERRγ deficiency through adenoviral-mediated overexpression of short-hairpin ERRγ (shERRγ) significantly decreased basal and alcohol-induced ERRγ and TFR2 mRNA levels in livers of mice (Figure 4G). Overall, these results demonstrate that ERRγ is a key transcriptional mediator of CB1 receptor-induced TFR2 gene expression.

### 2.4. TFR2 Is a Direct Target of ERRγ

**Figure 5:**
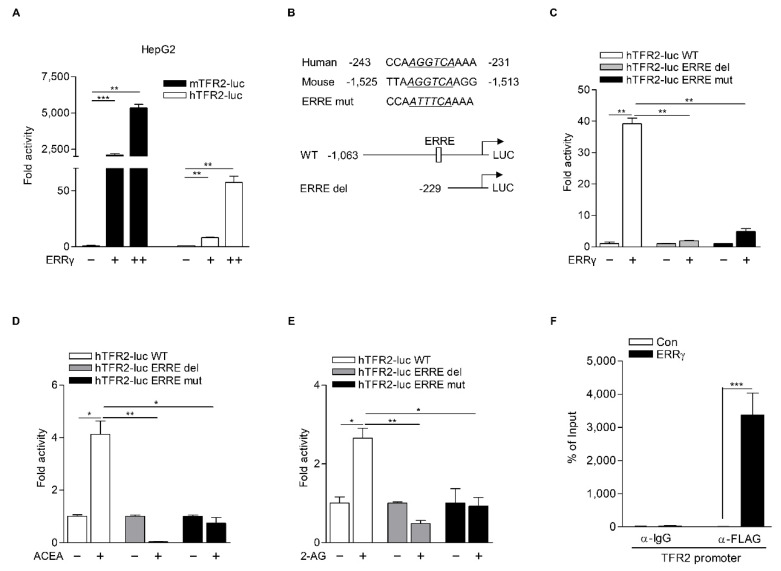
ERRγ directly upregulates TFR2 gene transcription. (**A**) ERRγ activates both mouse and human TFR2 gene promoter. HepG2 cells were transiently transfected with a luciferase reporter vector carrying mouse TFR2 promoter (mTFR2-luc) or human TFR2 promoter (hTFR2-luc) along with expression vector for ERRγ (+: 100 ng, ++: 300 ng) for 48 h. (**B**) Alignment of ERR-response element (ERRE) on human and mouse TFR2 promoter (*top panel*). ERRE sequences were italicized and underlined. Mapping of deletion constructs of hTFR2 promoter (*bottom panel*). WT: wild-type; del: deletion. (**C**) HepG2 cells were transfected with hTFR2-luc WT, hTFR2-luc del, or hTFR2-luc ERRE mut along with an expression vector for ERRγ (+: 300 ng) for 48 h. (**D**,**E**) HepG2 cells were transfected with hTFR2-luc WT, hTFR2-luc del, or hTFR2-luc ERRE mut for 48 h and then treated with ACEA (10 μM) (**D**) or 2-AG (10 μM) (**E**) for 6 h. (**F**) ChIP assay showing the occupancy of ERRγ on the ERRE of the hTFR2 promoter. HepG2 cells were transfected with expression vector for FLAG-ERRγ for 48 h. Soluble chromatins were then immunoprecipitated with an anti-IgG or an anti-FLAG antibody. Ten percent of soluble chromatins were used as input. All experiments were performed in duplicate (**A**,**C**–**E**) or triplicate (**F**) and repeated at least twice. Error bars show ± S.D. * *p* < 0.05, ** *p* < 0.01, *** *p* < 0.001 by two-tailed Student’s *t*-test.

Next, we explored molecular mechanisms involved in the regulation TFR2 gene transcription by ERRγ. We first examined whether ERRγ induced TFR2 promoter activity in HepG2 cells transfected with plasmids that carried human and mouse TFR2 promoters fused to luciferase and encoded ERRγ. As a result, ERRγ significantly increased both human and mouse TFR2 promoters (Figure 5A). It is reported that ERRγ could bind to extended half-site core sequences (TNAAGGTCA; ERR response element (ERRE)) as a monomer [31]. Close investigation of TFR2 promoter sequences revealed a putative ERRE motif (AGGTCA) conserved in human and mouse. Thus, we generated an ERRE mutant (hTFR2-luc ERRE mut) and an ERRE deletion construct (hTFR2-luc ERRE del) of human TFR2 promoter (Figure 5B). Transient transfection analysis performed in HepG2 cells showed that ERRγ significantly increased the activity of the wild-type TFR2 promoter (hTFR2-luc WT). Such effect was almost completely abolished by ERRE deletion or ERRE mutation of the TFR2 promoter (Figure 5C). Consistent with these findings, treatment with ACEA or 2-AG failed to increase the activity of hTFR2-luc ERRE del and hTFR2-luc ERRE mut (Figure 5D,E). A ChIP assay was also carried out using HepG2 cells. It demonstrated that ERRγ could strongly bind to ERRE of human TFR2 promoter (Figure 5F). These findings indicate that ERRγ can directly mediate CB1 receptor-induced TFR2 expression through its binding to the ERRE of the promoter of TFR2 gene.

## 3. Discussion

**Figure 6:**
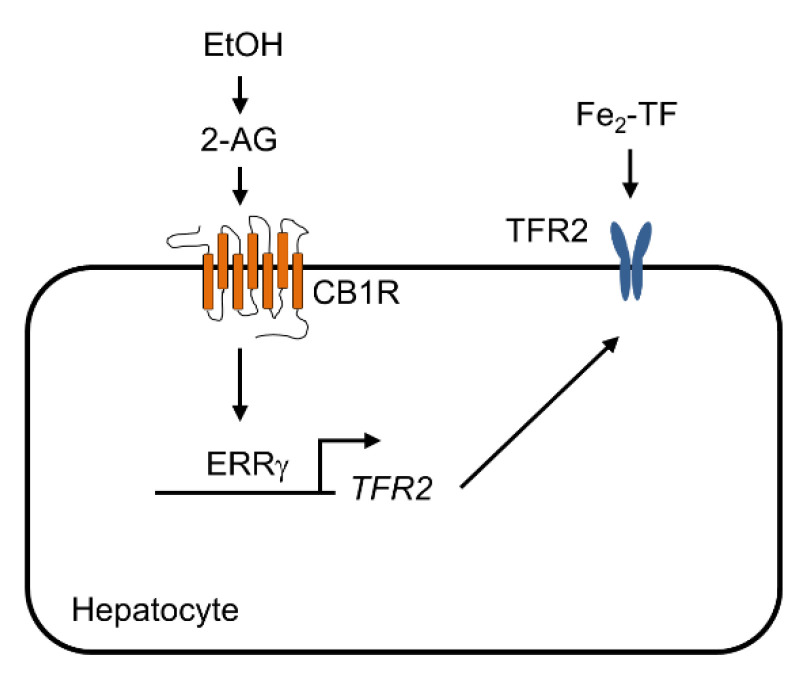
Proposed role of ERRγ in CB1 receptor-mediated TFR2 gene expression in hepatocytes. An endocannabinoid 2-AG increases ERRγ expression by activating CB1 receptor signaling in hepatocytes, subsequently resulting in induction of TFR2 gene expression which contributes to hepatic iron accumulation.

Alcohol is oxidized mainly in the liver by alcohol dehydrogenase and the cytochrome P450-dependent microsomal ethanol oxidizing system (MEOS), resulting in the production of acetaldehyde and ROS [32]. Previously, we reported that the orphan nuclear receptor ERRγ can regulate hepatic expression of CYP2E1, a major enzyme of MEOS, and promote oxidative stress and liver injury [33]. Interestingly, hepatic iron overload is observed in patients with ALD, while iron supplementation results in exacerbation of hepatocyte damage, liver fibrogenesis, and even cirrhosis in murine models [21,34,35]. These findings suggest that there is a causal relation between alcohol and hepatic iron overload, resulting in exacerbation of ALD. Indeed, it was reported that the TFR for importing iron into hepatocytes by receptor-mediated endocytosis of transferrin-iron complex plays a critical role in hepatic iron overload in patients with ALD [22]. However, the molecular mechanism underlying endocrine signaling and transcriptional regulation mediating alcohol-dependent TFR gene expression in hepatocytes is largely unknown. In this study, we demonstrated that endocannabinoid 2-AG could activate CB1 receptor signaling in hepatocytes, which in turn could increase ERRγ and TFR2 gene expression. Gain-of-function and loss-of function studies performed using cultured hepatocytes and mouse livers clearly showed that ERRγ could regulate TFR2 gene expression at a transcriptional level. These findings were further supported by results showing that ERRγ could directly bind to an ERRE of the promoter of the TFR2 gene, as demonstrated by deletion and mutation analyses of the promoter of the TFR2 gene in hepatocytes as well as by ChIP assay. Therefore, we conclude that ERRγ is a novel transcriptional regulator of CB1 receptor-mediated TFR2 gene expression (Figure 6).

Interestingly, we found that the induction ratio of TFR2 mRNA was different between HepG2 and AML12 cells, while the induction ratio of TFR2 mRNA by ACEA- or 2-AG treatment is comparable in these cell lines. HepG2 cells were derived from a hepatocellular carcinoma, while AML12 cells were derived from the livers of transgenic mice overexpressing transforming growth factor alpha [36]. Interestingly, it was reported that HepG2 and AML12 cells displayed different cellular responses in signal transduction and gene regulation [37]. For example, HepG2 and AML12 cells treated with insulin showed a different expression ratio of insulin receptor, insulin receptor substrate 1 (IRS1) and IRS2, a different activation ratio of Akt, and a different induction ratio of gluconeogenic genes, PCK1 and G6PC [37]. In addition, it is reported that the basal TFR2 expression was also different in HepG2 and Huh7 cells [38,39]. Therefore, the induction ratio of TFR2 expression by ACEA- or 2-AG treatment would be different between HepG2 and AML12 cells. It was reported that TFR2 overexpression in Huh7 cells involved a significant increase in cellular iron uptake and ferritin levels, which were reduced to control levels following TFR2 siRNA transfection [12]. Therefore, the induction ratio of TFR2 expression by ACEA- or 2-AG treatment would be different between HepG2 and AML12 cells. It was reported that TFR2 overexpression in Huh7 cells involved a significant increase in cellular iron uptake and ferritin levels, which were reduced to control levels following TFR2 siRNA transfection.

It was reported that ERRs are constitutively active due to their active conformation of LBD [40]. However, the ERRγ transcriptional activity seems to mainly depend of endocrine and metabolic signals such as glucagon, insulin, and endocannabinoids, at the transcriptional or post-transcriptional level. For example, ERRγ gene expression is induced by glucagon during fasting condition to stimulate the hepatic gluconeogenic program [31,41]. In addition, insulin can inhibit the transcriptional activity of ERRγ by Akt phosphorylation of ERRγ at Serine 179, eliciting translocation of ERRγ from the nucleus to the cytoplasm [28]. Moreover, hepatic ERRγ gene expression is induced by the alcohol-mediated activation of the CB1 receptor, resulting in CYP2E1-dependent oxidative liver injury [8]. In addition, unlike ERRγ, ERRα gene expression was not induced by glucagon or endocannabinoids in hepatocytes [8,31]. These findings suggest that ERRγ can act as an inducible transcriptional regulator and mediate endocrine and metabolic signals in hepatocytes. Interestingly, we found that all ERRs could significantly induce TFR2 mRNA expression in hepatocytes. However, we did not observe any significant change in the levels of ERRα and ERRβ in ACEA- or 2-AG treated and ERRγ-deficient hepatocytes, ruling out potential compensatory roles of ERRs in the regulation of TFR2 expression. It was also reported that both ERRα and ERRγ partly share their downstream target genes critical for oxidative capacity and ion homeostasis in peripheral tissues such as skeletal and cardiac muscles, kidney, and stomach [23]. This is not surprising considering that ERRs share a nearly identical DNA-binding domain and that their transcriptional activities depend on their interactions with similar coregulator proteins. On the other hand, ERRα can inhibit effects of ERRγ and PGC-1α on hepatic gluconeogenesis. Moreover, the Akt phosphorylation site in ERRα has not been found yet [28,31,42]. Unlike ERRγ, ERRα does not increase the activity of CYP2E1 or hepcidin gene promoter in hepatocytes [8,29]. These results suggest that the transcriptional regulation and post-translational modification of ERRs would be critical for their different transcriptional and physiological outcomes. The molecular mechanism for a crosstalk between membrane receptor and each ERR isoform needs further characterization.

In conclusion, we demonstrate that ERRγ plays a critical role in CB1 receptor-mediated TFR2 gene expression in hepatocytes. The findings of this study revealed a previously unrecognized role of ERRγ in not only a crosstalk among membrane receptors, CB1 receptor, and transferrin receptor, but also a causal relationship between ALD and hepatic iron overload.

## 4. Materials and Methods

### 4.1. Chemicals

ACEA (arachidonyl-2′-chloroethylamide) and 2-AG (2-arachidonyl glycerol) were purchased from Tocris Bioscience (Ellisville, MO, USA). GSK4716 was purchased from Abcam (Abcam, Cambridge, UK). All chemicals were dissolved in the recommended solvent.

### 4.2. Plasmid DNAs and Recombinant Adenoviruses

Promoters of human TFR2 (−1063/+38) and mouse TFR2 (−2220/+38) were PCR amplified from human and mouse genomic DNAs and then cloned into the *Mlu* I/*Xho* I portion of the PGL3-control vector. The hTFR2-luc ERR del (−229/+38) was cloned into the *Mlu* I/*Xho* I portion of the PGL3-control vector by PCR with hTFR2-luc WT (−1063/+38) as a template. hTFR2-luc ERRE mut (−240 bp AGGTCA −234 bp to −240 bp ATTTCA −234 bp) was generated using an EZchange Site-directed Mutagenesis Kit (Enzynomics, Daejeon, Korea). Expression vectors for ERRα, ERRβ, and ERRγ were described previously [26]. All plasmids used were confirmed by sequence analysis. Ad-green fluorescent protein (Ad-GFP), Ad-ERRγ, Ad-US (unspecific short hairpin), and Ad-shERRγ were described previously [8,29]. All viruses were purified using CsCl_2_.

### 4.3. Cell Culture, Transfection and Luciferase Assay

HepG2 (human liver cancer cell line; ATCC, Manassas, VA, USA) cells and Huh7 (human hepatoma cell line, ATCC) cells were cultured in Dulbecco’s modified Eagle’s medium (DMEM, high glucose, Welgene, Gyeongsangbuk-do, Korea) and RPMI 1640 medium (Welgene) supplemented with 10% fetal bovine serum and 1% antibiotics (penicillin-streptomycin). AML12 (mouse hepatocyte cell line, ATCC) cells were cultured in DMEM/F-12 medium (Welgene) containing an additional 40 ng/mL dexamethasone and 1% insulin-transferrin-selenium-pyruvate supplementation under the same conditions as other cells. These cells were grown in an incubator with 5% CO_2_ at 37 °C. Transient transfection was performed using Superfect (QIAGEN, Hilden, Germany) and polyethylenimine (Polysciences, Inc., Warrington, PA, USA) transfection reagent in accordance with each manufacturer’s instructions. Cells were used for experiments at 80% confluence. An appropriate amount of empty vector was added to adjust total DNA amount used for each transfection to be 1 μg/well and co-transfected Nano as an internal control. Firefly luciferase activity was normalized to Nano-Glo (Promega, Madison, WI, USA) luciferase activity. ACEA and 2-AG were used to treat at a concentration of 10 μM for 12 h. GSK4716 was treated in HepG2 and AML12 cells at a concentration of 10 μM for 12 h. Luciferase activity was measured at 48 h after transfection.

### 4.4. Animal Experiment

C57BL/6J mice (The Jackson Laboratory, Bar Harbor, Maine, USA) were used for all animal experiments. For gain-of-function studies, Ad-GFP (5 × 10^9^ pfu) and Ad-Flag-ERRγ (5 × 10^9^ pfu) were injected through tail veins of 8-week-old mice (*n* = 3 per group). Mice were sacrificed at 6 days after the injection. For loss-of-function studies, Ad-US or Ad-shERRγ was injected via tail veins of male C57BL/6J mice (*n* = 5 per group). At day 6 after the injection, mice were orally administered vehicle or alcohol (6 g/kg) for 24 h. All animal experiments were approved by the Institutional Animal Care and Use Committee of the Korea Research Institute of Bioscience and Biotechnology (KRIBB-AEC-18104). All experimental procedures for mice were performed in accordance with the Guide for the Care and Use of Laboratory Animals published by the US National Institutes of Health.

### 4.5. Quantitative Real-Time PCR Analysis

Total RNAs were isolated from HepG2 cells and AML12 cells after transfection or chemical treatment using a Tri-RNA Reagent (Favorgen Biotech Corporation, Ping-Tung, Taiwan) according to the manufacturer’s instructions. cDNAs generated using a TOPscript RT DryMIX dT18plus (Enzynomics) were analyzed using a CFX Connect real-time system (Bio-Rad, Hercules, CA, USA) with TOPreal qPCR 2X PreMIX (Enzynomics). Samples were amplified for 40 cycles at 95 °C for 10 s, 60 °C for 15 s, and 72 °C for 15 s. Relative gene expression levels were analyzed using the ΔΔCt method. All primers were listed in Supplementary Table S1.

### 4.6. Protein Extraction and Western Blot Analysis

Whole cell extracts were extracted using RIPA buffer (Thermo Fisher Scientific, Waltham, MA, USA). Extracted proteins were separated on a 10% SDS polyacrylamide gel electrophoresis gel and transferred to a nitrocellulose membrane (GE Healthcare, IL, USA). The membranes were probed with anti-FLAG (Invitrogen, Carlsbad, CA, USA, diluted 1:2000), anti-β-actin (Santa Cruz Biotechnology, Dallas, TX, USA, diluted 1:3000), anti-hTFR2 (Santa Cruz Biotechnology, 1:1000 dilution), anti-mTFR2 (Invitrogen, 1:1000 dilution), and anti-ERRγ antibodies (1:2000 dilution). Anti-ERRγ antibody was generated using a peptide (404-AGQHMEDPRRAGKMLM-419) from mouse ERRγ helix 9 [28]. The primary antibody was detected using an HRP-conjugated secondary antibody and an ECL kit (GE Healthcare) according to the manufacturer’s instructions. Images were obtained using a ChemiDoc XRS system (Bio-Rad).

### 4.7. Chromatin Immunoprecipitation (ChIP) Assay

ChIP assay was performed using a SimpleChIP Plus Enzymatic Chromatin IP Kit (Cell Signaling Technology, MA, USA) according to the manufacturer’s protocol. Immunoprecipitation was performed using an anti-FLAG M2 affinity gel (Sigma-Aldrich, St. Louis, MO, USA) or IgG as a negative control (Cell Signaling Technology). After DNA was recovered, quantitative real-time PCR (Q-PCR) was performed using primers targeting part of the human TFR2 promoter. Primers used for q-PCR were listed in Supplementary Table S1.

### 4.8. RNA Interference

AML12 cells were transfected with control (si-Con, 25 nM) and mouse ERRγ (si-ERRγ, 25 nM) siRNAs using Lipofectamine RNAi MAX (Thermo Fisher Scientific) according to the manufacturer’s instructions. si-Con and si-ERRγ were purchased from Bioneer (Bioneer, Daejen, Korea).

### 4.9. Statistical Analysis

Results are expressed as means ± S.D. All statistical analyses were carried out using the two-tailed Student’s *t*-test (GraphPad Prism 3 software). Differences were considered statistically significant at *p* < 0.05.

## Data Availability

The authors declare that the data supporting the findings of this study are available within the paper and its supplementary information files. Raw data is available from the corresponding author upon reasonable request.

## Supplementary Materials

The following are available online at https://www.mdpi.com/article/10.3390/ijms22116021/s1, Table S1: qPCR primer sequences.
