# Supplementary material for: Orphan Nuclear Receptor ERRγ Is a Transcriptional Regulator of CB1 Receptor-Mediated TFR2 Gene Expression in Hepatocytes
Source: Int J Mol Sci. 2021 Jun 2;22(11):6021. doi: 10.3390/ijms22116021 (PMC8199698; doi:10.3390/ijms22116021)
Supplement: Supplementary file 1 [file ijms-22-06021-s001.zip › ijms-1213929-supplementary.pdf]

Supplementary Table S1. qPCR primer sequences.

Primer Name	Forward (5′-3′)	Reverse (5′-3′)
L32	TCTGGTGAAGCCCAAGATCG	CTCTGGGTTTCCGCCAGT
ERRα	AGGAGTACGTCCTGCTG	CCTCAGCATCTTCAATG
ERRβ	GGACTCGCCGCCTATGTTC	CGTTAAGCATGTACTCGCATTTG
hmERRγ	AAGATCGACACATTGATTCCAGC	CATGGTTGAACTGAATTCCCAC
ChIP-hTFR2	CAGGAGAGGGGAGGGGGCAC	CCCCGGGTCCAGGCCAGAGC
hTFR2	GGTGACCAATGCTCAGGACTT	CAGGTGTGTAGGGGTCTCCA
mTFR2	TGGTCCAAGATATCCTCGAT	CCGGTGGCGCTGTAGGGACA
hTFR1	GTAAACTGGTCCATGCTAAT	TAACAATGGGAAATTTAGTC
mTFR1	GGTAAACTGGTCCATGCTAA	CAAGGTCTGCCTCAACAACG
